# Phenomics and its potential impact on livestock development in low-income countries: innovative applications of emerging related digital technology

**DOI:** 10.1093/af/vfaa002

**Published:** 2020-04-01

**Authors:** Raphael Mrode, Chinyere Ekine Dzivenu, Karen Marshall, Mizeck Gift Gibson Chagunda, Bridgit Syombua Muasa, Julie Ojango, Ally Mwai Okeyo

**Affiliations:** 1 Livestock Genetics Program, International Livestock Research Institute, Nairobi, Kenya; 2 Animal and Veterinary Science, Scotland Rural College, Roslin Institute Building, Easter Bush, UK; 3 Animal Breeding and Husbandry in the Tropics and Subtropics, University of Hohenheim, Stuttgart, Germany; 4 Supporting Evidence-Based Interventions, The Royal (Dick) School of Veterinary Studies and The Roslin Institute, Easter Bush Campus, UK

**Keywords:** dairy cattle, emerging digital technologies, information and communications technology, novel traits

**Implications**

- Data capture systems in Sub-Saharan Africa and India for dairy cattle and small ruminants have been based on infrastructure similar to those in developed countries and have mainly been unsuccessful.
- The application of information and communications technology, mobile phones, and other digital innovations has shown some promising results but reliable internet connectivity still poses a challenge.
- Animal scientists will need to collaborate with an interdisciplinary team of scientists to design the next generation of innovative technologies which are cheap, robust, easy to use, and can function without internet connectivity for efficient capture of performance data in Sub-Saharan Africa.
- Application of emerging technologies will be critical to attracting youth into agriculture in developing countries and thus ensuring sustainable phenomics platforms.

## Introduction

Phenotypes play an important role in understanding the genetic basis of livestock performance and are vital in informing and ensuring effective herd and flock management. At the national level, capturing phenotypes is fundamental in national aggregates of production data that underlines government agricultural policies and projections. Therefore, phenomics is important at both the farm level for profitability and at the national level for effective government agricultural policies. Phenomics may be regarded as the application of technologies to enable the collection of phenotypes cheaply, easily, and in large volumes. This may be driven by the need for automation enabling high throughput collection of phenotypes or to enable indirect collection of phenotypes which are difficult and expensive to measure through affordable and high throughput innovations. In Sub-Saharan Africa, with agricultural systems based on subsistence farming and small holder systems, capturing phenotypes has always been a major challenge. This could be attributed to several factors including 1) production systems characterized with many small farms and few animals, therefore the inability of farmers to pay for the cost of recording phenotypes, 2) small farms that are highly dispersed making the logistics of recording very cost inefficient and unattractive to private investors, and 3) the lack of government support for such services (Chagunda et al., 2006; Kosgey and Okeyo, 2007; Visser and van Marle‐Köster, 2017). Therefore, earlier attempts to capture phenotypes have mostly been restricted to the easy to measure traits such as body weight (either measured directly or predicted) and milk yield. However, fitness traits such as fertility, resilience to local environments, and survival have largely been ignored.

## Examples of Historical Attempts to Capture Data in Some Developing Countries

Some early attempts to capture phenotypes have tended to mimic systems in developed countries, with less regard to the fundamental differences between the two systems. These attempts often led to failures but these have been successful in some cases. A few of these early attempts to capture data are briefly summarized below.

### Examples of recording activities in small ruminants

Some historical attempts to capture phenotypes for growth traits in sheep and goats in some countries (e.g., India and Kenya) have been associated with research projects aimed at implementing breed improvement programs. Examples include the goat improvement program involving 34 villages by the Nimbkar Agricultural Research Institute in 1991 in South-Central Maharashtra of India with the aim of improving productivity in goats through cross-breeding (Nimbka, 1999). The Sirohi bucks involved in the cross-breeding were selected using records on their individual growth rates and their mothers’ milk yields. However, the project collapsed after a few years including the performance recording due to lack of funds. Second, the Kenya Dual Purpose Goat Development Project which was started in 1980 with the aim of developing a synthetic breed of goat that combined the adaptability of the indigenous East African and Galla goats and the growth and milk producing abilities of the Toggenburg and Anglo-Nubian breeds (Ojango et al., 2010). The project ran for several years with on-station testing in a nucleus herd and on-farm testing by several farmers with milk yield recorded. However, at about 2005, there were only a few animals involved in the project mostly due to funding issues leading to termination of the project and recording activities.

However, in countries such as South Africa where several goat breed associations were established and in Kenya where the Meru goat breeder association was formed as part of the FARM-Africa dairy goat and animal healthcare project this has led to a more sustainable system of capturing various performance data for goats (Ahuya et al., 2009; Visser and Van Marle-Köster, 2017). A summary of the traits recorded by some of the goat breeders’ association in South Africa is presented in Table 1. For instance, the Angora Goat Breeders’ Society was established in 1892, the South African Boer Goat Association in 1959, and the South Africa Milch Goat Breeders’ Society was formed in 1958. The existence of these breed association meant that phenomics was not only restricted to “the easier to capture traits” such as growth but linear type traits were also recorded. In the case of Angora goat, traits such as fiber diameter and fleece weight were also captured and selected for (Visser and Van Marle-Köster, 2014).

**Table 1.** A summary of goat breed societies in South Africa and their recording activities

Goat breed societies	Year formed	Examples of traits recorded
Angora Goat Breeders’ Society	1892	Fleece weight, fiber diameter, comfort factor (%) and spinning effective fineness
The South Africa Milch Goat Breeders’ Society	1958	Milk yield, milk composition and linear type traits
South African Boer Goat Association	1959	Birth weight, weaning weight, weaning rate, growth rate, kidding rate (kids born or does mated) and twining rate

### Examples of performance recording in dairy cattle

Mrode (2019) presented a detailed historic perspective for the establishment of milk recording services in several African countries, India, and Brazil. In some African countries (Kenya and Zimbabwe) and in South American countries (Brazil and Argentina), phenomics aimed at capturing milk records in the dairy sector in the early 1900s were initiated by stud books or breed associations with government support and/or funding from international development agencies. Usually these milk recording activities were conﬁned to large herds owned by settler farmers in these countries (Kosgey et al., 2011). The traits focused on were milk yield and milk solids (fat and protein percent). Most of these records were associated with far too complex institutional arrangements and high costs; hence most of these systems collapsed when project and government support were withdrawn (Kosgey et al., 2011) or they were able to transit successfully to schemes where farmers pay for such services usually accompanied with an initial drop in number of farms recoded as was the case in Kenya and Brazil (Trivedi, 1998; Costa et al., 2004). Currently, these systems are still operating in these two countries with farmers paying for the recording activities with little or no government support. Milk recording in India has, however, been based on a slightly different approach with the National Dairy Development Board working in collaboration with several developmental agencies and Non-Governmental Organizations such as the Bharatiya Agro Industries Foundation (**BAIF**) providing these services. However, as mentioned by Ducrocq et al. (2018), the recording is immature with a high percentage of the records of limited use due to poor quality such as unknown sire, animal identiﬁcation errors, or transcription mismatches when entering information in the database.

## Current or Modern Trends in Phenomics in Developing Countries

**Figure 1.**
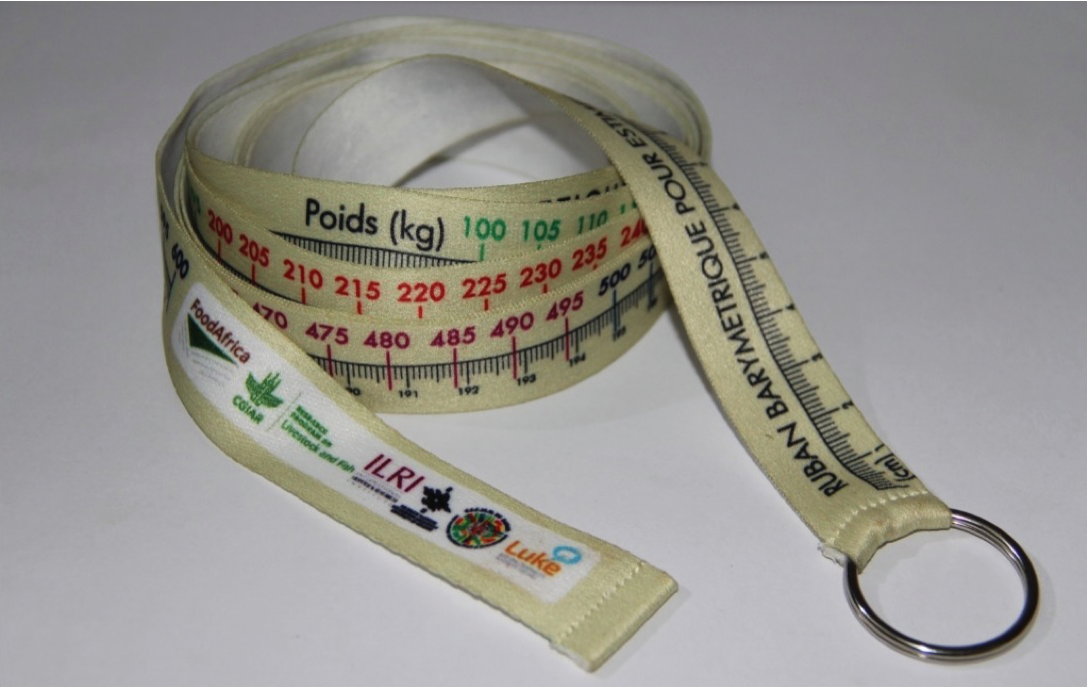
Tape for measuring heart girth of animals for the prediction of body weight.

The production systems of small holder farmers characterized with small and dispersed herds in addition with associated high cost of performance recording constitute some of the bottlenecks to sustainable phenomics in developing countries. Therefore advances in mobile technology has prompted attempts to investigate information and communications technology (**ICT**) models for performance recording in small holder systems and for the feedback of management information to farmers to help them make informed decisions. A summary of some of the initiatives on the application of ICT and mobiles phones and other digital tools for data collection in some developing countries is presented in Table 2. Some of the projects applying modern technologies for data capture include the African Dairy Genetic Gain (**ADGG**) and the Private Public Partnership for AI Delivery (**PAID**) sponsored by the Bill and Melinda Gates Foundation in Tanzania and Ethiopia for the capture of performance records (milk yield), hearth girth for the prediction of body weight, body condition score, and insemination data for dairy cattle. The performance and fertility data were collected monthly by using a software based on the Open Data Kit, installed in tablets as well as on mobile phones employing the services of performance recording agents. Heart girth has been measured in animals using a tape (Figure 1) for the indirect prediction of body weight. Moreover, a technological platform called iCow (http://www.icow.co.ke/), owned by a private company called Green Dreams, was used as means of feeding back management information to farmers and for their training. The achievements of the project as of March 2018 are summarized at https://www.slideshare.net/ILRI/adgg-achievement and selected results presented in Table 3. The performance data collected from ADGG has enabled genomic prediction and selection of top young bulls for breeding (Mrode et al., 2019). Similarly, Ducrocq et al. (2018) examined the use of ICT for the collection of performance data on a large scale in India by the BAIF. The project consisted of 170 AI technicians equipped with multi-component software, installed ﬁrst on dedicated “data loggers” and later on mobile phones. The outcome was a rapid collection of hundreds of thousands of good quality fertility records; however, the quality of milk production data was not as good.

**Table 2.** Summary of digital tools that have been employed for capturing performance data in some developing countries

Initiatives	Livestock	Tools	Traits recorded	Countries
African Dairy Genetic Gains Project	Dairy cattle	Mobile phones and tablets Open data kit, information, and technology Platform called iCow (http://www.icow.co.ke/)	Milk yield, body condition score, heart girth and insemination details	Ethiopia, Tanzania, Kenya
Dairy Project Bharatiya Agro Industries Foundation (BAIF), India	Dairy cattle	Data loggers and mobile phones	Milk yield	India
Community-Based Breeding Program	Sheep and goats	Digital system, aniCloud (https://anicloud.com/), and software aniCapture	Birth weight, body weight at various ages and twinning rate	Ethiopia, Malawi
Muasa (2020)	Dairy cattle	Pedometers	Cow activities for prediction progesterone profile (fertility)	Kenya

**Table 3.** Number of farmers and animals registered and monitored on the African Dairy Genetics Gains (ADGG) platform by October 2019

Country	Registered		Monitored	
	Farmers	Animals	Farms	Animals
Ethiopia				
ADGG	12,576	36,042	6,559	19,658
PAID	50,460	60,944		
Tanzania				
ADGG	15,690	38,914	13,589	26,433
PAID	18,585	23,170		
Total	97,311	159,070	20,148	46,091

PAID = Public private partnership for AI delivery.

In the case of small ruminants, the implementation of data collection in a group of farmers through the Community-Based Breeding Programs (**CBBP**) in Ethiopia and Malawi (Table 2) have resulted in successfully capture of performance data related to growth traits, twinning rate, and fleece weight (Haile et al., 2019). In addition, a digital system, AniCloud (https://anicloud.com/), which integrates with AniCapture, a smart device software designed for offline gathering of data in situations where connectivity is challenging has been employed for data collection. The CBBP underscores the importance of farmer participation and use of ICT tools in the successful collection of performance data.

Capturing fitness traits in small holder systems constitute a major challenge as it is more involving compared with measuring growth traits. However innovative approaches are emerging for the capture of novel phenotypes on fertility. Some of these approaches include estrus detection through the use of sensors to detect the different behavior patterns (time and duration) of the animal and generate famer-useful information. This may involve the use of electronic rump-, neck-, or leg-mounted detectors that transmit data via either radio signal or ultra-wide band technology to a receiver or video cameras to supplement visual observations (Bruyere et al., 2012). An increasingly popular method for estrus detection is through measurement of activity through the use of pedometers. Cows in heat tend to be restless resulting in increased movement (Baxter et al., 1977) and the sensors, usually attached to the hind leg, record the number of steps made by the cow per unit of time. Measures of cow activity are then used to predict progesterone profile for individual cows. Application of such activity sensors have been attempted in small holder farms in Kenya. In a recent study involving a large scale farm in UK and Kenya, and small holder dairy farms in Kenya, Muasa (2020) demonstrated very comparable results in the sensitivity and specificity of three different estrus detection technologies (rapid progesterone, CowAlert, and Estrotect).

## Future Perspective for Phenomics in Developing Countries

The widespread use of mobile phones and rapidly advancing ICT presents novel opportunities for innovative approaches to data capture. The ADGG and CBBP have demonstrated feasibility of such approaches. However, this emphasizes the need to design and develop simple, efficient, inexpensive, non-evasive, and sturdy phenotyping devices to support collection of a range of existing or new quantitative data relating to animal welfare, production, reproduction, product quality, feed efficiency, etc. on the farm. Most suitable technologies will be those that will support routine data collection and near to real-time transmission to agile databases, with robust analytics that enable meaningful feedback to be sent to users (researchers, producers, policy makers, etc.) as quickly as possible, and which can be easily incorporated into genetic improvement. However, reliable internet connectivity has been a challenge in the application of some of these digital tools for data capture in some countries shown in Table 2. Animal scientists will need to collaborate with an interdisciplinary team of scientists (software developers, engineers, programmers, etc.) capable of designing a variety of innovative technologies needed for innovative phenotypic data collection. A major emphasis will be technological systems that can work offline in data capture and transmission, as reliable internet connectivity is a challenge in many developing countries.

Preliminary attempts of innovative approaches to capture fertility traits have been described in a previous section. Such methods and other emerging cheap and innovative approaches such as the use of mid-infrared spectrum for indirect prediction of various economically important traits in dairy cattle will need to be calibrated and adapted for use in small holder systems. While measuring feed intake remains an expensive undertaking even in developing countries, the use of automatic systems to capture feed intake (e.g., the Insentec Roughage Intake Control system, the GrowSafe system, or the Calan Broadbent Feeding System), in a set up mimicking small holder systems to evaluate the impact of locally available feed resources on performance or to derive some predictive equations will not only provide useful guidelines to farmers, but may also be useful in providing approximate measures of feed intake, which might be better than the current situation with no information on feed intake

Development and application of innovative and efficient methods of data collection will generate a large amount of data from herds or flocks located in different places, and if geo-referenced will enable such data to be linked with related global meta-weather and soil data, thus further enriching the dataset. The amount of stored information will be substantial, so an important aspect of phenomics in developing countries is the need for efficient data infrastructure for permanent storage as well as software and web applications that allow easy access and analysis of these data by stakeholders. Designed data bases or platforms need to be secure and should be able to: 1) easily interface with other databases and 2) accommodate integrated compilation of phenotypic and genotypic data to support generation of substantial sample sizes needed for rigorous data analysis to inform management practices and optimize animal production systems. Increasing the volume of genomic and production data collected on individual animals across production environments will enhance the ability to select animals for desired performance traits suited to specific agro-ecological areas. Use of such data for management decisions by farmers might encourage them to undertake the expense and labor necessary to collect the needed data.

Currently, various government and international policies exist that govern the movement and use of germplasm across countries. However, with digital tools offering more opportunities to collect performance data from small holder systems (including farmers’ location), similar policies might be needed to govern the use of such data.

## Conclusion

In summary, elaborate infrastructure, herds of small sizes which are widely dispersed plus the high cost of recording have been some of the bottlenecks to sustainable phenotyping systems in developing countries in Sub-Saharan Africa. In spite of these challenges, advances in technology and innovative use of ICT and mobile technologies to capture performance data have been demonstrated for dairy cattle and small ruminants. However, reliable internet connectivity continues to be the main challenge. To a small extent, use of digital sensors to indirectly capture “the not-too easy to capture” traits have been tested with promising prospects. Animal scientists will need to collaborate with an interdisciplinary team of scientists to design the next generation of innovative technologies which are cheap, robust, easy to use, and can function without internet connectivity for efficient capture of performance data in developing counties. Emerging and innovative approaches for measuring traits directly and indirectly need to be calibrated and adapted to the conditions prevailing in small holder systems. Application of these technologies will be critical to attracting youth into agriculture in developing countries and thus ensuring sustainable phenomics platforms.

## About the Authors

**Raphael Mrode** holds a joint position as a Professor of Quantitative Genetics and Genomics at the Scotland Rural College (SRUC), United Kingdom and Principal Scientist in Quantitative Dairy Cattle Genetics at the International Livestock Research Institute (ILRI), Kenya. He obtained his PhD from the University of Edinburgh in Animal Genetics and Breeding. He has been involved in development and research underpinning genetic evaluations in dairy and beef cattle in the United Kingdom for over 20 yr. He is a member of the Technical Committee of INTERBULL and is an auditor of the International Committee for Animal Recording (ICAR). His research interest is in the development and application of statistical methods and linear models for national and international genetic evaluations, genomic selection and modelling of novel traits in dairy cattle. **Corresponding author**: raphael.mrode@sruc.ac.uk

**Chinyere (Ekine) Ekine-Dzivenu** is a statistical geneticist at the International Livestock Research Institute (ILRI) in Kenya. She is using animal genomics, improved animal genetics and breeding strategies to aid sustainable development and reduce poverty and hunger in developing countries. She completed her PhD and a Postdoctoral fellowship at Livestock Gentec in Alberta, Canada. Prior to that, she completed her Master of Science in Quantitative Genetics and Genome Analysis at the University of Edinburgh.

**Karen Marshall** has been working at the International Livestock Research Institute (ILRI) as a research scientist within the 'utilisation of animal genetic resources' group. She leads the project 'Senegal Dairy Genetics' which aims to identify which breed / cross-breed of dairy animals is most appropriate for smallholder farmers in Senegal. She is also involved with projects in Uganda, Somalia, The Gambia, Mali, Guinea, Senegal and Vietnam. She has a special interest in the genetics underpinning disease resistance. Prior to this position she was employed as a senior lecturer within the School of Rural Science and Agriculture at the University of New England in Armidale, Australia.

**Mizeck Gift Gibson Chagunda** is a Professor and Chair of Animal Breeding and Husbandry in the Tropics and Subtropics at the University of Hohenheim, Germany. Mizeck earned his doctorate degree in Animal Breeding and Genetics from the University of Goettingen, Germany, after obtaining an MSc and BSc from the University of Malawi. He previously worked at the University of Malawi, Aarhus University in Denmark, and SRUC (Scotland’s Rural College) in Scotland. Mizeck’s research interests are in improving biological and economic efficiency in livestock production systems. He achieves this through investigating novel phenotypes and difficult-to-measure traits; livestock’s environmental impact, and the use of technologies, data-driven decision support systems, and optimizing animal breeding strategies and breeding goals.

**Bridgit Syombua Muasa** is a Doctor of Veterinary Medicine from the University of Nairobi in Kenya. She is actively involved in Cosmos Education, child rights activism and youth empowerment, development and leadership initiatives in Kenya and is a volunteer of the Kenya Red Cross Society.

**Julie Ojango** is an Animal Genetics and Breeding scientist working at the International Livestock Research Institute (ILRI) who has more than ten years of continuous advancement in livestock genetic improvement in developing countries, and a proven track record of applying appropriate practical mix of technologies that meet the industry needs. Prior to working at ILRI, she was a senior lecturer of animal sciences at Egerton University in Kenya. She has excellent skills in livestock data management and quantitative analyses, and is well versed in dairy cattle and small ruminant production systems in developing countries.

**Ally Mwai Okeyo** is a Principal Scientist in the ILRI’s Global Livestock Genetics- Live Gene Research Program. Okeyo is a quantitative geneticist with over 30 years of experience in practical design and implementation of livestock improvement programs in sub-Saharan Africa and South Asia. Okeyo previously led ILRI’s Breeding strategies Research, specifically focusing on development and implementation projects, covering a wide range of research areas including, characterization and genetic diversity of indigenous tropical livestock, their improved utilization, as well as development and application of assisted reproductive technologies in dairy cattle.

## References

[CIT0001] Ahuya C.O., Okeyo A.M., Mosi R.O., Peacock C., and Ojango J.M.K.. 2009 Performance of Toggenburg dairy goats in smallholder production systems of the eastern highlands of Kenya. Small Ruminant. Res. 8:7–13. Doi:10.1016/j.smallrumres.2008.11.012

[CIT0002] Baxter S.E., King G.J., and Hurnik J.F.. Studies related to the use of exteroceptive stimuli, pedometers and vaginal probe as estrus detection aids. Dairy Ind. Res. Rep. Univ. Guelph; 1977; p. 62–63.

[CIT0003] Bruyère P., Hétreau T., Ponsart C., Gatien J., Buff S., Disenhaus C., Giroud O., and Guérin P.. 2012 Can video cameras replace visual estrus detection in dairy cows? Theriogenology 77:525–530. doi:10.1016/j.theriogenology.2011.08.027. 22137603

[CIT0004] Chagunda M.G.G., Msiska A.C.M., Wollny C.B.A., Tchale H., and Banda J.W.. 2006 An analysis of smallholder farmers’ willingness to adopt dairy performance recording in Malawi. Livest. Res. Rural Dev. 18(5):66.

[CIT0005] Costa C.N., Teixeira N.M., Freitas A.F., Cobuciand J.A., and Haguihara K.. 2004 Trends in milking recording in Brazil. In: Performance recording of animals: State of the art, 2004 Proceedings of the 34th Biennial Session of ICAR, Volume 11; May 28–June 3, 2004; Sousse, Tunisia: EAAP Scientific Series.

[CIT0006] Ducrocq V., Laloe D., Swaminathan M., Rognon X., Boichard M.T., and Zerjal T.. 2018 Genomics for ruminants in developing countries: from principles to practice, Front. Genet. 9:694. doi:10.3389/fgene.2018.00251. 30057590 PMC6053532

[CIT0007] Haile A., Gizaw S., Getachew S., Joaquín T., Mueller P., Amer P., Rekik M., and Rischkowsky B.. 2019 Community‐based breeding programmes are a viable solution for Ethiopian small ruminant genetic improvement but require public and private investments. J. Anim. Breed and Gen. 136:319–328. doi:10.1111/jbg.12401 31037758

[CIT0008] Kosgey I.S., Mbuku S.M., Okeyo A.M., Amimo J., Philipsson J., and Ojango J.M.. 2011 Institutional and organizational frameworks for dairy and beef cattle recording in Kenya: a review and opportunities for improvement. Anim. Genet. Res. 48:1–11. doi:10.1017/S2078633610001220.

[CIT00009] Kosgey I.S., and Okeyo A.M. 2007 Genetic improvement of small ruminants in low input, smallholder: technical and infrastructural issues. Small Ruminant Res. 70:76–88.

[CIT0009] Mrode R 2019 Genetic and genomic dairy cattle evaluations in developing countries. In: van der Werf J., and Pryce J., editors. Advances in breeding of dairy cattle. Cambridge (UK): Burleigh Dodds Science PublishingLimited https://shop.bdspublishing.com/store/bds/detail/workgroup/3-190-83625

[CIT0010] Mrode R., Aliloo H., Ekine C., Ojango J., Gibson D.J.P., and Okeyo M.. 2019 The application of several genomic models for the analysis of small holder dairy cattle data. Proceedings of the 2019 Interbull Meeting. Interbull Bull. 55:70–76 https://journal.interbull.org/index.php/ib/article/view/1486

[CIT0011] Muasa B.S 2020 Monitoring the reproductive status of dairy cows using cow-side oestrus detection technologies [PhD Thesis]. University of Edinburgh.

[CIT0099] Nimbka C 1999 A village goat cross-breeding project in Maharashtra, India. Workshop for developing breeding strategies for lower input Animal Production environments. Bella, Italy, September, 1999. ICAR Technical Series 3, 435–443 [accessed February 5, 2020]. https://www.icar.org/Documents/technical_series/ICAR-Technical-Series-no-3-Bella/Nimbkar.pdf.

[CIT00012] Ojango J.M.K., Okeyo A.M., and Rege J.E.O. 2010 The Kenya dual purpose goat development project. Animal Genetics Training Resource Case Study. Nairobi, Kenya: ILRI [accessed February 2, 2020]. https://cgspace.cgiar.org/handle/10568/3743.

[CIT0012] Trivedi K.R 1998 International workshop on animal recording for smallholders in developing countries. ICAR Technical Series—No 1 [accessed February 5, 2020]. https://www.icar.org/index.php/publications-technical-materials/technical-series-and-proceedings/icar-technical-series-by-single-contribution/icar-technical-series-1/.

[CIT0013] Visser C., and Van Marle-Köster E.. 2014 Strategies for the genetic improvement of South African angora goats. Small Ruminant Res. 121:89–95.

[CIT0014] Visser C., and van Marle‐Köster E.. 2017 The development and genetic improvement of South African Goats. In Goat Science Sándor Kukovics, IntechOpen. doi: 10.5772/intechopen.70065 [accessed November 29, 2019]. Available from: https://www.intechopen.com/books/goat-science/the-development-and- genetic-improvement-of-south-african-goats.

